# Extraction, structural characterization, and biological activities of a new glucan from *Codonopsis pilosula*

**DOI:** 10.1038/s41598-023-31660-2

**Published:** 2023-03-18

**Authors:** Shanshan Lu, Wei Gu, Qihan Ma, Rong Tian, Rongli Qiu, Lijie Ma, Yinzhi Wu, Mengxue Liu, Junjie Tang

**Affiliations:** 1 School of Pharmacy, Nanjing University of Chinese Medicine, Nanjing, 210023 China; 2 Jiangsu Collaborative Innovation Center of Chinese Medicinal Resources Industrialization, Nanjing University of Chinese Medicine, Nanjing, 210023 China; 3 Hanlin College, Nanjing University of Chinese Medicine, Taizhou, 225300 China; 4 Suzhou TCM Hospital Affiliated to Nanjing University of Chinese Medicine, Suzhou, 215008 China

**Keywords:** Drug discovery, Plant sciences, Health care, Chemistry

## Abstract

In this study, a powerful and rapid aqueous two-phase system (ATPS) method was used to extract polysaccharides from *Codonopsis pilosula*. The ATPS process was investigated with response surface methodology (RSM). At an ammonium sulfate concentration of 17%, ethanol concentration of 30%, and extraction temperature of 40 °C at pH 6, the total extraction yield of polysaccharides reached (31.57 ± 1.28)%. After separation and purification, a homogenized polysaccharide CPP 2–4 with molecular weight of 3.9 × 10^4^ kDa was obtained from the bottom phase. The physicochemical properties and structural features confirmed that CPP 2–4 was an α-1,6-glucan. Activity studies showed that the IC_50_ of CPP 2–4 for DPPH radical scavenging was 0.105 mg/mL. The FRAP and ABTS assays showed that CPP 2–4 had strong antioxidant activity in a dose-dependent manner. Furthermore, CPP 2–4 inhibited NO release in RAW264.7 cells induced by lipopolysaccharide, which indicated a certain anti-inflammatory effect. This study improved the extraction rate of polysaccharides from *C. pilosula* and identified a glucan for the first time, that can contribute to a better understanding of the composition and structure of polysaccharides from *C. pilosula* and provide data support for the medicine and food homology of *C. pilosula*.

## Introduction

*Codonopsis pilosula*, a well-known Chinese traditional medicine, has been officially listed in the Homologous Catalogue of Medicine and Food, and the 2020 edition of “Chinese Pharmacopoeia”, where it is recorded as strengthening the spleen, tonifying the lungs, nourishing the blood, and engendering liquid^1^. The main components of the dried root of *C. pilosula* include sugars, polyacetylenes, alkaloids and nitrogenous compounds, flavonoids, phenylpropanoids, and volatile oil^2–4^. Literature studies have shown that *C. pilosula* polysaccharides (CPPs) have different pharmacological effects, including regulating gastrointestinal function, improving lung function, regulating immunity, anti-tumor effects, and enhancing hematopoietic function. Based on the available papers, there are 48 polysaccharides have been isolated from different parts of *C. pilosula.* Only 27 CPPs’ chemical structures or linkages have been resolved at present^5,6^. CPPs have attracted extensive attention owing to their relative nontoxicity, and lack of side effects, residue, and tolerance^7–10^. However, current research on CPPs has limitations, including limited extraction methods, incomplete composition research, and low polysaccharide yields (highest rate of 22.3%^11^). CPPs can be obtained by water extraction and alcohol precipitation^12–19^, ultrasound-assisted extraction^20,21^, microwave-assisted extraction^22,23^, enzymatic hydrolysis extraction^24,25^, subcritical water extraction^26^, and other methods. However, some of these methods are time-consuming and cumbersome, or have high instrument requirements, such that they cannot be widely used.

Recently, a novel liquid–liquid extraction method, the aqueous two-phase system (ATPS) technique, has been applied to the separation of plant polysaccharides in a single-step procedure. ATPS refers to two types of insoluble aqueous phase systems spontaneously formed by mixing aqueous solution of two hydrophilic compounds in a certain mass fraction. Similar to the traditional solvent extraction principle, the selective distribution of target substances between the two phases might be related to intermolecular hydrogen bonds, van der Waals forces, hydrophobicity, interface properties, and other factors^27^. Owing to its advantages, such as short separation time, low viscosity, high extraction efficiency, and no environmental pollution, ATPS is currently widely used for the extraction of biomolecules, phenylethanoid glycosides, and various biological products, such as cells, nucleic acids, proteins, and amino acids^28,29^. However, its application to CPP extraction has not yet to be reported. In this study, the ATPS system was used to extract CPP, which is expected to solve the problem of the low extraction rate and provide a basis for further CPP research and applications.

## Materials and methods

### Materials and reagents

The medicinal materials in this study were the cultivated *C. pilosula*, which were harvested during the harvest period. They were identified as *Codonopsis pilosula* (Franch.) Nannf by Professor Gu Wei of Nanjing University of Chinese Medicine. The voucher samples and digital image information were stored in the Herbarium of Nanjing University of Chinese Medicine.

Dried roots of *C. pilosula* were purchased from Pingshun County, Shanxi province, China. The herbs were dried at 50 °C, cut into 1 cm strips, and crushed into powder that passed through a sieve with 80 mesh. Mannose, rhamnose, glucuronic acid, galacturonic acid, glucose, galactose, xylose, arabinose, fucose (all ≥ 98.0% purity), papain, and 1-phenyl-3-methyl-5-pyrazolone (PMP) were purchased from Shanghai Yuanye Bio-Technology Co., Ltd. Trifluoroacetic acid (TFA) and other chemicals were purchased from Shanghai Macklin Biochemical Co., Ltd. Acetonitrile (HPLC grade) was obtained from Merck Darmstadt Ltd. (Germany). All chemicals were reagent grade or better.

### Preparation of samples

**Figure 1:**
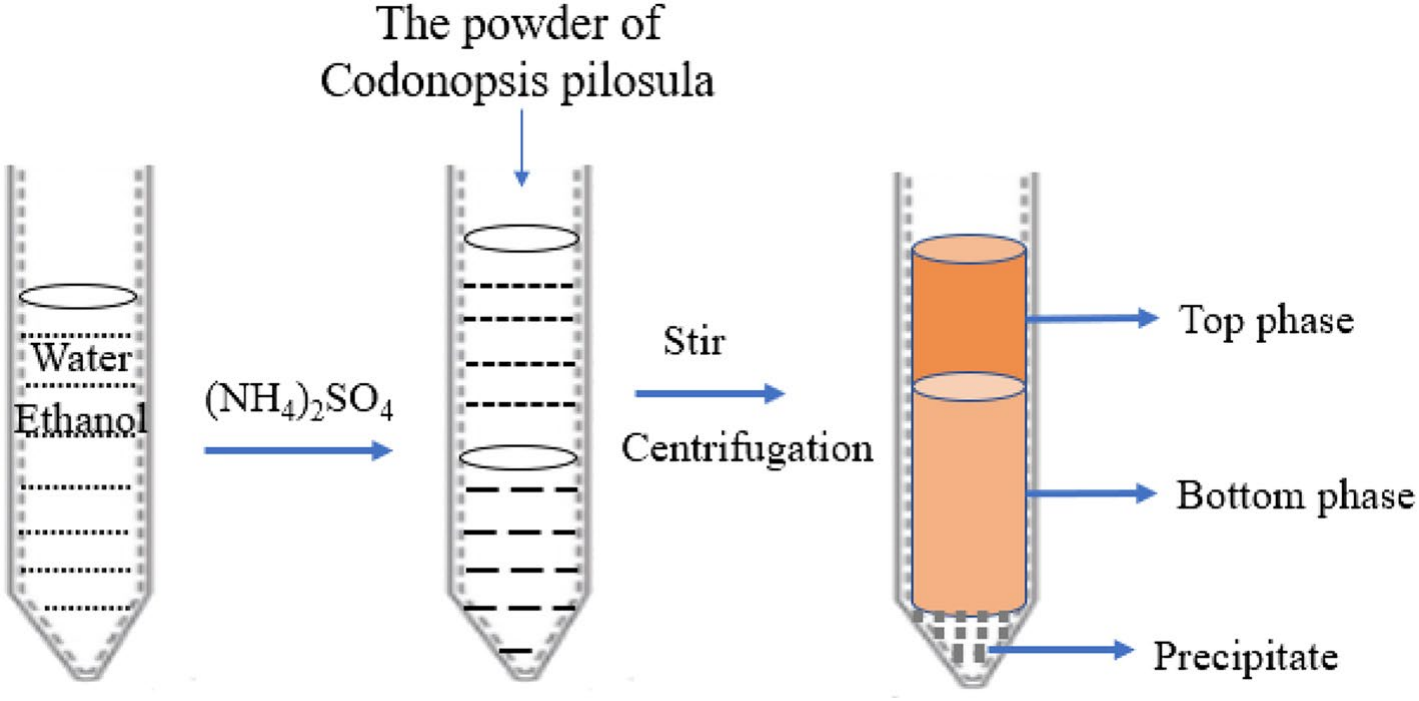
Process of simultaneous extraction and separation of polysaccharides by the ATPS method.

CPP was extracted using the ATPS method. *C. pilosula* powder was refluxed with petroleum ether (the boiling point is 60–90 °C) for 6 h at 80 °C to remove pigment and lipid compounds from the sample, and the solids were separated and dried for later use. As shown in Fig. 1, an ethanol/ammonium sulfate system was prepared by dissolving ammonium sulfate in distilled water and adding an appropriate amount of ethanol, to which the solids (0.5 g, accurately weighed) were added. The system was fully stirred and ultrasonic for 30 min, and the solution was centrifuged at 4000 rpm for 10 min to separate the two phases completely^29^. The volumes of the top and bottom phases were recorded and the yields (Y) were calculated. The polysaccharide concentrations in the two-phase solution were determined by the PhOH–H_2_SO_4_ method using the following equations:

$${Y}_{t}\left(\%\right)=100{C}_{t}{V}_{t}/({C}_{t}{V}_{t}+{C}_{b}{V}_{b})$$

$${Y}_{b}\left(\%\right)=100{C}_{b}{V}_{b}/({C}_{t}{V}_{t}+{C}_{b}{V}_{b})$$

where V_t_ and V_b_ are the volumes (mL) of the top and bottom phases, respectively, C_t_ and C_b_ are the polysaccharide concentrations in the top and bottom phases (μg/mL), and Y_t_ and Y_b_ are the polysaccharide yields in the top and bottom phases, respectively.

### Single-factor investigation of ATPS extraction

Initially, different amounts of ammonium sulfate (concentrations of 13%, 14%, 15%, 16%, 17%, 18%, 19%, and 20%, maintaining a total weight of 10 g) were added, while the other relevant variables were fixed (ethanol concentration, 30% (w/w); pH 6; temperature, 25 °C). Next, using the optimal ammonium sulfate concentration, different concentrations of anhydrous ethanol (22.5%, 25%, 27.5%, 30%, 32.5%, maintaining a total weight of 10 g) were added, while the other relevant variables were fixed (pH 6; temperature, 25 °C). Next, using the optimal concentrations of ammonium sulfate and ethanol, and with the other relevant variable fixed (temperature, 25 °C), hydrochloric acid solution (0.1 mol/L) was used to adjust the system pH to 3.0, 3.5, 4.0, 5.0, 6.4, with the total mass maintained at 10 g. Finally, using the optimal ammonium sulfate and ethanol concentrations, and pH, different system temperatures (10, 20, 30, 40, and 50 °C) were tested by heating in a water bath.

The polysaccharide yields in the top and bottom phase solutions were determined according to the method described in section "Preparation of samples".

### Optimization of CPP extraction process using response surface methodology

To obtain the optimal extraction process, the Box–Behnken design (BBD) was used to determine the process parameters. According to these principles, a three-factor and three-level experimental design was established (Table 1). Statistical analysis was performed using the Design-Expert version 8.05 software package (Stat-Ease, Minneapolis, MN, USA). A quadratic polynomial model was defined to fit the response (polysaccharide yield, %):

$$Y={\beta }_{0}+\sum_{i=1}{\beta }_{i}{X}_{i}+\sum_{i=1}^{n}{\beta }_{ii}{{X}_{i}}^{2}+\sum_{i=1}^{n}\times \sum_{j>1}^{n}{\beta }_{ij}{X}_{i}{X}_{j}$$

where *β*_0_, *β*_i_, *β*_ii_, and *β*_ij_ are the intercept coefficient, linear term, quadratic term, and interaction term, respectively, and X_1_ (ammonium sulfate concentration), X_2_ (ethanol concentration), and X_3_ (temperature) are coded variables ranging from − 1 to + 1.

**Table 1:** Response surface factor level and encoding.

Factor	Code	Level		
		− 1	0	1
Ammonium sulfate, %	A	16	17	18
Ethanol, %	B	27.5	30	32.5
Temperature, °C	C	30	40	50

### Separation and purification of CPP 2–4

**Figure 2:**
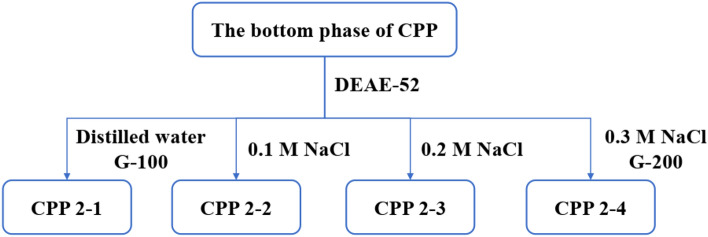
Purification flowchart of CPP 2–4 from the bottom phase.

After depigmentation and protein removal, the obtained bottom phase was placed in a dialysis bag (M_w_, 500 Da) for 24 h, freeze-dried, and stored. An appropriate concentration of the bottom-phase polysaccharide solution was prepared, as shown in Fig. 2. A DEAE-52 cellulose column was used for separation and purification, eluting with distilled water, 0.1 M NaCl, 0.2 M NaCl, and 0.3 M NaCl, respectively. After dialysis (M_w_, 10,000 Da) and proper concentration, the elution site was further purified using dextran gel G-200. The eluted site was collected, dialyzed for 24 h, and freeze-dried to obtain CPP 2–4.

### Homogeneity and molecular weight determination

The homogeneity and molecular weight of CPP 2–4 were determined by high-performance gel permeation chromatography (HPGPC) using ELSD 3300 detector and TSKgel amide-80 column (5 μm, 4.6 mm × 25 cm, TOSOH). Glucans of known molecular weight (T10, T40, T50, T70, T100) were used as reference materials and distilled water was used as the mobile phase. The flow rate was 1.0 mL/min and the column temperature was 35 °C. CPP 2–4 was dissolved in mobile phase (1 mL), centrifuged at 12,000 rpm for 10 min, and the supernatant (20 μL) was injected for detection. The drift tube temperature was 110 °C.

### Structural characteristics of CPP 2–4

#### Monosaccharide composition

The monosaccharide composition of CPP 2–4 was determined by HPLC with precolumn derivatization. In brief, TFA (4 mL, 4 mol/L) was added to CPP 2–4 (4 mg), and the resulting product was hydrolyzed at 110 °C for 4 h to remove excess acid and then dissolved in distilled water (0.2 mL). NaOH solution (0.1 mL, 0.6 M) and PMP methanol solution (0.2 mL, 0.5 M) were added to the above solution, which was then vortexed and heated in a water bath at 70 °C for 100 min. Next, HCl (0.2 mL, 0.3 M) was added to neutralize the solution, followed by trichloromethane (1 mL). After mixing thoroughly, the mixture was centrifuged at 12,000 rpm for 10 min, and then trichloromethane (1 mL) was added to the supernatant. This operation was repeated three times. Finally, the supernatant was fixed to a volume of 2 mL and filtered through 0.22-μm microporous filter membrane and analyzed by HPLC (Agilent 1260 Infinity chromatograph) with a UV detector at 254 nm.

The analytical column was C_18_ (5 μm, 4.6 mm × 250 mm, Agilent, USA) and operated at 30 °C. Mobile phase A was acetonitrile and mobile phase B was 0.1 mol/L phosphate buffer (pH 7.65). The gradient program was as follows: 0–28 min, 17% A; 28–40 min, linear gradient to 30% A; 40–45 min, 30% A; 45–50 min, linear gradient to 17% A. Elution was performed at a flow rate of 1.0 mL/min and the injection volume was 10 μL.

#### Fourier transform infrared (FT-IR) spectroscopy

The infrared spectrum of CPP 2–4 was obtained by FT-IR spectroscopy (Bruker Tenson 37, Germany). An appropriate amount of polysaccharide powder was directly measured in the range of 4500–500 cm^−1^.

#### Nuclear magnetic resonance (NMR) spectroscopy

NMR spectra were obtained using a Bruker 500 NMR spectrometer. CPP 2–4 (30 mg) was dissolved in heavy water (D_2_O, 0.5 mL) and lyophilized. This procedure was repeated three times to exchange deuterium. ^1^H, ^13^C, and 2D (^1^H–^1^H COSY, HSQC, and HMBC) NMR spectra were obtained.

### Bioactivity of CPP 2–4

#### Antioxidant activity of CPP 2–4

The antioxidant activity of CPP 2–4 was determined using a previously reported method of measuring the 2,2-diphenyl-1-picrylhydrazyl (DPPH) free radical scavenging activity, with some modifications^28^. CPP 2–4 solutions (1.0 mL) of different concentrations (0.05, 0.1, 0.2, 0.4, 0.6, 0.8, and 1.0 mg/mL) were added to DPPH–ethanol solution (1.0 mL, 100 μM) and allowed to react in the dark at room temperature for 30 min. The absorbance was measured at 517 nm using a UV-2550 spectrophotometer^30,31^.

The ferric reducing antioxidant power (FRAP) was determined according to the method of specification, which was modified for a 96-well microplate reader. The FRAP assay measures the ability of antioxidants in CPP 2–4 to reduce ferric-tripyridyl-triazine (Fe^3+^-TPTZ) complex to the blue ferrous form (Fe^2+^), which absorbs light at 593 nm. Briefly, standard or sample extract (5 μL) was mixed with FRAP solution (180 μL, prepared by mixing TPTZ diluent, TPTZ solution, and detection buffer in a ratio of 10:1:1 (v/v/v)) and added to the wells. The plate was incubated at 37 °C for the duration of the reaction. Absorbance readings were taken at 593 nm using a UV–vis microplate kinetic reader (Infinite M200pro, Tecan). CPP 2–4 solutions of different concentrations (0.05, 0.1, 0.2, 0.4, 0.6, 0.8, and 1.0 mg/mL) were assayed to determine the antioxidant activities.

2,2'-Azino-bis(3-ethylbenzothiazoline-6-sulfonic acid) (ABTS) is oxidized to green radical ABTS^•+^ in the presence of appropriate oxidants. ABTS^•+^ production is inhibited by antioxidants. Briefly, standard or sample extract (10 μL) and peroxidase (20 μL) were mixed with ABTS solution (170 μL, prepared by mixing 1/1000 H_2_O_2_ solution, ABTS solution, and detection buffer in the ratio of 1:19:1.25 (v/v/v)), added to the 96 wells, and blended. Absorbance readings were taken at 414 nm using a UV–vis microplate kinetic reader (Infinite M200pro, Tecan). Trolox standard solutions at five different concentrations (0.15, 0.3, 0.6, 0.9, 1.2, and 1.5 mM) were prepared to form a standard curve. The total reducing ability of CPP 2–4 solutions of different concentration (0.05, 0.1, 0.2, 0.4, 0.6, 0.8, and 1.0 mg/mL) were expressed using the trolox equivalent antioxidant capacity (TEAC).

#### Anti-inflammatory activities of CPP 2–4

##### Cytotoxicity assays^32–34^

Mouse macrophage cell line RAW264.7 cells were cultured in Dulbecco's modified Eagle medium (DMEM; containing 10% fetal bovine serum (FBS), 100 U/mL penicillin, and 100 U/mL streptomycin) in an incubator with 5% CO_2_ at 37 °C. RAW264.7 cells in the logarithmic phase with good growth status were seeded into 96-well cell culture plates at a density of 1 × 10^5^ cells/mL, and cell suspension (100 μL) was added to each well and cultured in the incubator for 24 h. After removing the supernatant, DMEM solutions containing different concentrations of CPP 2–4 (0.01, 0.1, 1, 10, and 100 μg/mL) were added, with DMEM solution also directly added to the blank control group. Six wells were repeated for each sample. After incubation for 24 h and 48 h, 3-(4,5-dimethylthiazol-2-yl)-2,5-diphenyltetrazolium bromide (MTT) solution (20 μL) was added to each well, and the operation was kept out of light. The supernatant was removed after 4 h of incubation and dimethyl sulfoxide (DMSO, 150 μL) was added, continuing to avoid light. After crystals were completely dissolved in the culture plate, the absorbance value was measured at 490 nm using a microplate reader and the proliferation rate was calculated.

##### Detection of NO release from RAW264.7 cells using the Griess method

NO production was determined based on the amount of nitrite, a stable end product of NO, present in the conditioned medium using the Griess reaction. Briefly, RAW264.7 cells in the logarithmic phase with a good growth state were seeded into 96-well cell culture plates. CPP 2–4 (12.5, 25, 50, and 100 μg/mL) was added when the supernatant was removed after 24 h, and lipopolysaccharide (LPS) at a final concentration of 1 μg/mL was added for 24 h. DMEM solution was added to the blank group. The Griess method was used to detect NO production at 540 nm.

### Statistical analysis

The data were analyzed by GraphPad Prism 9, one-way ANOVA, and Design-Expert version 8.05 software. The results are presented as means ± SD. * means *P* < 0.05 and ** means *P* < 0.01, which were regarded as statistically significant.

### Ethical approval

It is hereby declared that the experimental methods and experimental materials involved in this paper were in compliance with relevant institutional, national and international guidelines and legislation.

## Results and discussion

### Optimization of CPP extraction conditions

**Figure 3:**
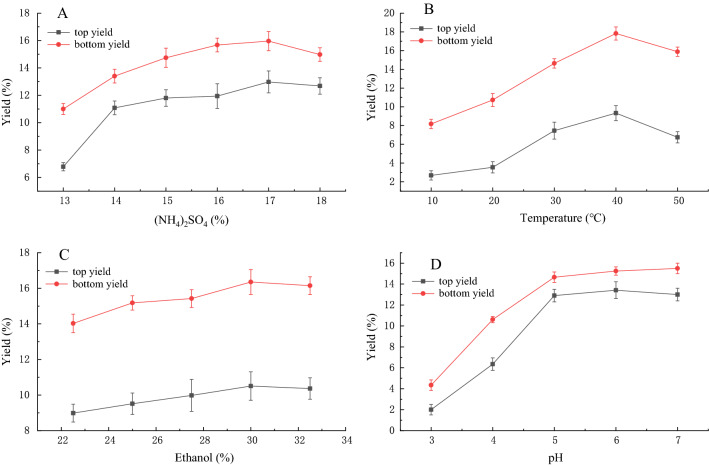
Effect of (**A**) ammonium sulfate concentration, (**B**) extraction temperature, (**C**) ethanol concentration, and (**D**) pH on polysaccharide extraction yield in the ATPS method.

As shown in Fig. 3(A) and (C), the polysaccharide yield in the top and bottom phases improved with increasing ammonium sulfate concentration in the range of 13–20%. Similarly, an increasing yield was observed in the ethanol concentration range of 22.5–32.5%. These results showed that the use of 17% ammonium sulfate and 30% ethanol gave a higher polysaccharide extraction rate. Extraction temperature and pH were also key factors affecting the extraction polysaccharide rate. Different temperatures and pH values were used in experiments, with the results shown in Figs. 3(B) and (D). The highest polysaccharide extraction rate was obtained at 40 °C and pH 6. The detailed data were shown in Tables S1–4.

According to the Box–Behnken experimental design principle, a three-factor and three-level experimental design was established in Table 1. According to the results of integrated single factor experiments, the ATPS conditions were optimized using the CPP yield as the response value as shown in Table 2. The fitted quadratic multinomial regression equation was *Y* = 30.9 + 0.16*A* + 0.63*B* + 0.95*C* − 0.05*AB* − 0.3*AC* − 0.73*BC* − 3.50*A*^*2*^ − 2.48*B*^*2*^ − 2.09*C*^*2*^, *R*^*2*^ = 0.9986. The value of Prob > F was < 0.0001, which was less than 0.05, showing that the model was significant. In the equation, B, C, BC, A^2^, B^2^, C^2^ were significant model parameters shown in Table 3.

**Table 2:** Response surface experiment design and data processing.

No	Ammonium sulfate, %	Ethanol, %	Temperature, °C	Polysaccharide yield, %	Predicted
1	17	32.5	50	27.45	27.19
2	18	30	50	25.76	25.66
3	17	27.5	50	27.64	27.38
4	18	32.5	40	25.76	26.12
5	16	27.5	40	23.97	24.08
6	17	32.5	30	26.48	26.74
7	17	30	40	31.57	30.90
8	16	30	50	26.25	26.41
9	17	30	40	30.63	30.90
10	17	30	40	30.49	30.90
11	16	32.5	40	25.34	25.44
12	18	30	30	24.97	24.81
13	16	30	30	24.27	23.91
14	17	30	40	30.73	30.90
15	17	30	40	31.09	30.90
16	18	27.5	40	24.59	24.49
17	17	27.5	30	23.76	24.02

**Table 3:** Regression analysis results.

Source of variation	Quadratic	Degree of freedom	Average deviation	F value	P-Prob > F
Regression model	119.66	9	13.30	67.35	< 0.0001
A	0.2	1	0.20	0.99	0.3530
B	3.21	1	3.21	16.28	0.0050
C	7.26	1	7.26	36.77	0.0005
AB	0.01	1	0.01	0.051	0.8284
AC	0.35	1	0.35	1.79	0.2224
BC	2.12	1	2.12	10.72	0.0136
A2	51.68	1	51.68	261.81	< 0.0001
B2	25.97	1	25.97	131.56	< 0.0001
C2	18.32	1	18.32	92.81	< 0.0001
Residual	1.38	7	0.20	–	–
Lack of fit	0.63	3	0.21	1.11	0.4440
Pure error	0.75	4	0.19	–	–
Total deviation	121.04	16	–	–	–
Index of correlation	0.9886	–	–	–	–

**Figure 4:**
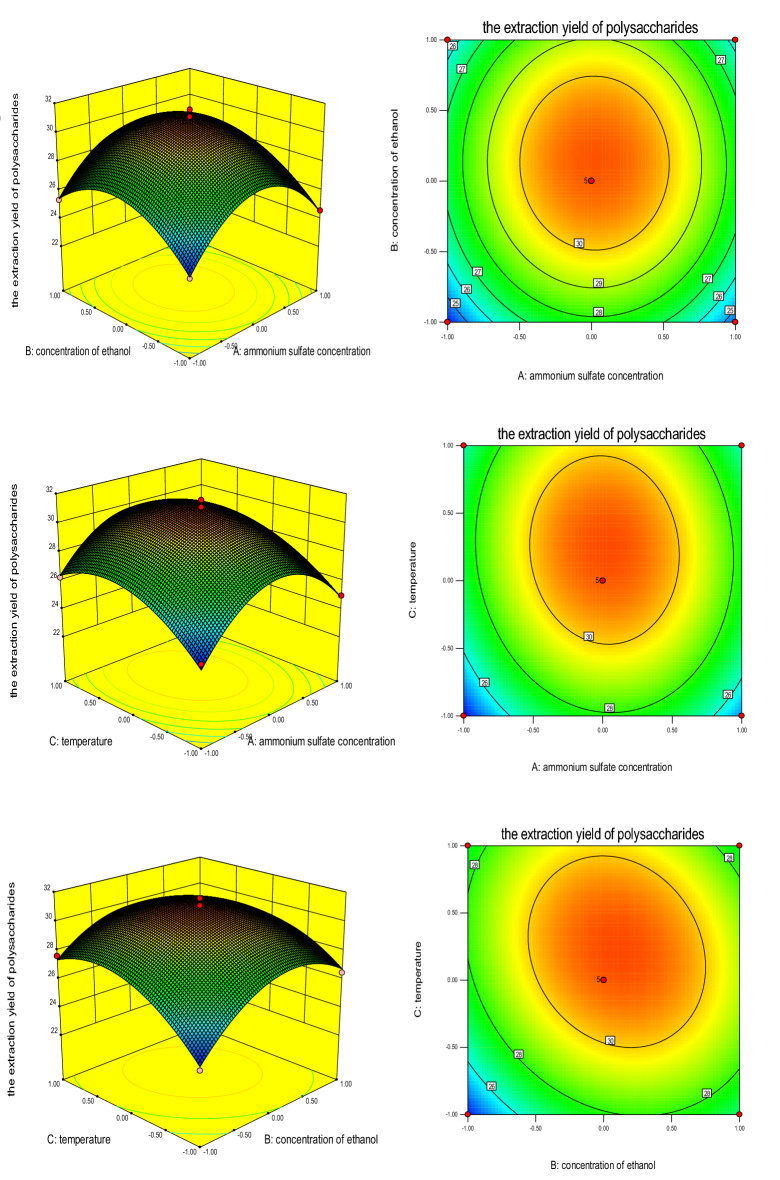
Effect of (**A**) ammonium sulfate concentration, (**B**) ethanol concentration, and (**C**) temperature on CPP extraction yield.

In summary, the results (Fig. 4) showed that the optimal extraction conditions for ATPS were as follows: Ammonium sulfate concentration, 17%; ethanol concentration, 30%; and temperature, 40 °C. Under these conditions, the CPP yield was up to (31.57 ± 1.28) %.

### Purification, homogeneity, and composition of CPP 2–4

**Figure 5:**
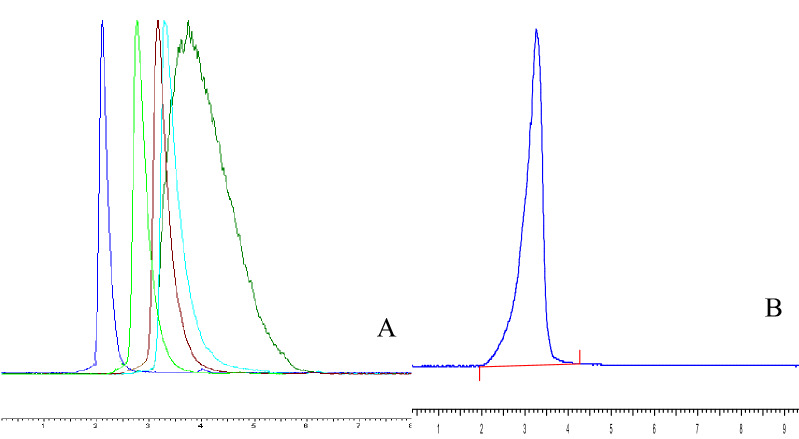
(**A**) HPGPC profiles of different dextrans, (**B**) HPGPC profile of CPP 2–4 (in **A**, the sequence of peaks from left to right is: Dextran 10, Dextran 40, Dextran 50, Dextran 70, Dextran 100).

**Figure 6:**
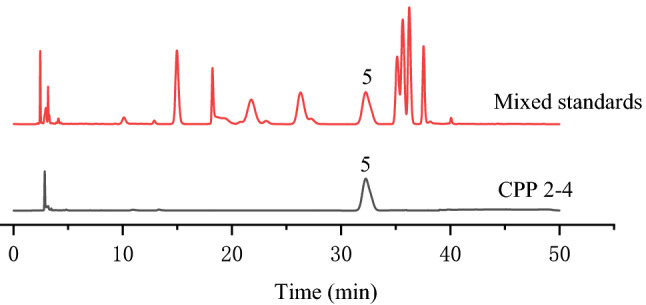
Monosaccharide composition of CPP 2–4; 5-glucose.

The top and bottom phases were successfully isolated from dried *C. pilosula*, affording yields of 11.3% and 20.27%, respectively. Owing to the better antioxidant activity of the bottom phase compared with the top phase, polysaccharides in the bottom phase were separated and purified. Purification of CPP 2–4 was finally achieved using DEAE-52 cellulose and Sephadex G-200 columns, eluting with 0.3 M NaCl solution, as shown in Fig. 2. The CPP 2–4 yield was (12 ± 1.2) %, the sugar content was (98 ± 1.3) %, and the protein content was (2.05 ± 0.06) %. The standard curve of different molecular weight glucans was *Y* =  − 6 × 10^−6^*x* + 1.4215 (*r* = 0.9985). As shown in Fig. 5, the M_W_ of CPP 2–4 was 3.9 × 10^4^ kDa, and its molecular weight distribution had the characteristics of polydispersion, indicating a homogeneous polysaccharide. HPLC–precolumn derivatization was performed to further analyze the composition of CPP 2–4. After precolumn derivatization by PMP, the monosaccharide composition of the hydrolysates was determined by HPLC. As shown in Fig. 6, the results showed only one glucose component in the HPLC diagram, which preliminarily determined that CPP 2–4 was a dextran.

### Structural characterization of CPP 2–4

**Figure 7:**
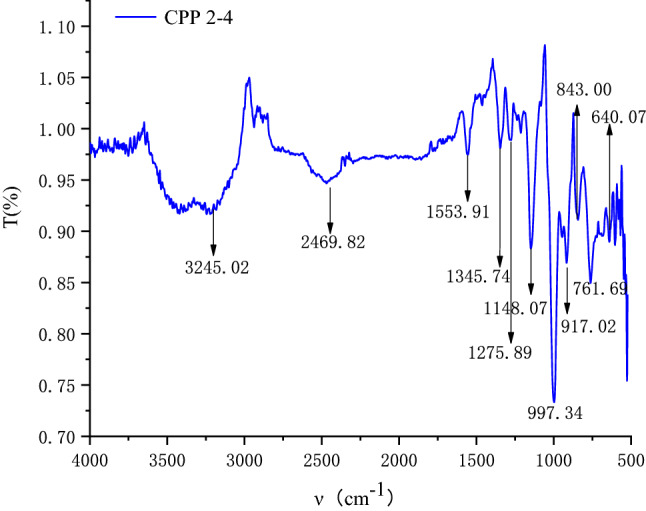
IR spectra of CPP 2–4.

To investigate the structure of CPP 2–4, films prepared with dried CPP 2–4 powder were subjected to FT-IR at 4000–500 cm^−1^. As shown in Fig. 7, the broad band between 3600 and 3200 cm^−1^ was characteristic of the hydroxyl group stretching vibration. The absorption band at 1148.07 cm^−1^ indicated the presence of a C–O stretching vibration^34^. Furthermore, the band at approximately 1345.74 cm^−1^ was attributed to the C–H stretching vibration. No absorption was observed at 1735 cm^−1^, indicating that CPP 2–4 did not contain glucuronic acid and that the sample was a neutral polysaccharide^35,36^. The structure of CPP 2–4 was preliminarily determined to be a dextran, which required further verification by NMR technology.

**Figure 8:**
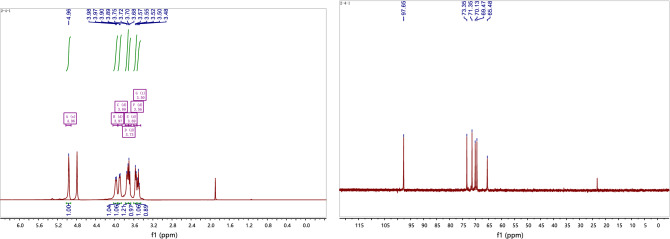
^1^H-NMR and ^13^C-NMR spectra of CPP 2–4.

**Figure 9:**
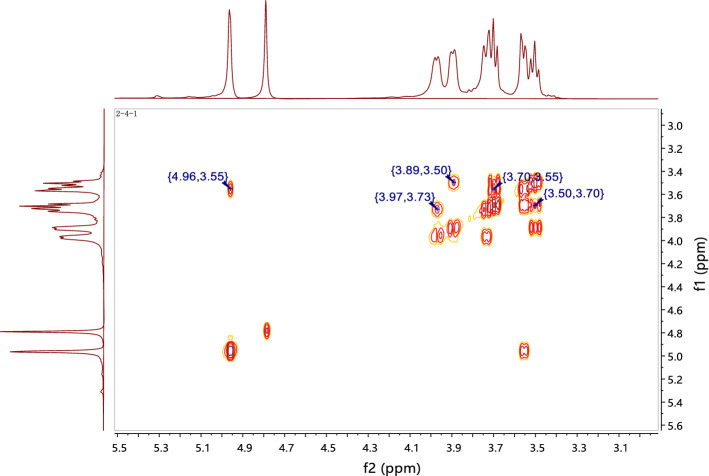
^1^H–^1^H COSY spectrum of CPP 2–4.

**Figure 10:**
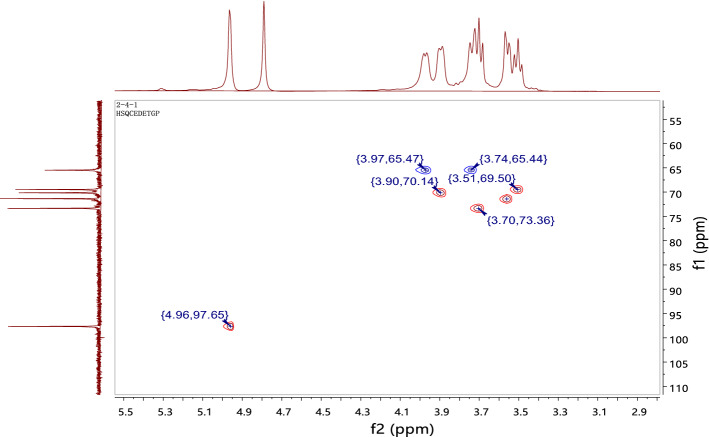
HSQC spectrum of CPP 2–4.

**Figure 11:**
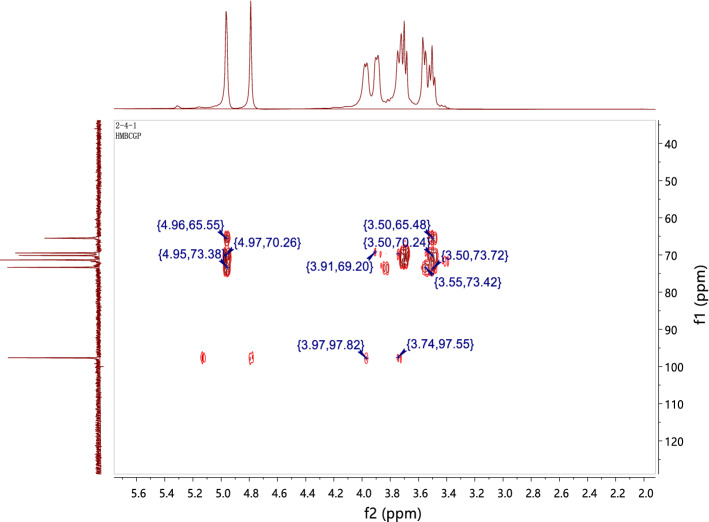
HMBC spectrum of CPP 2–4.

**Figure 12:**
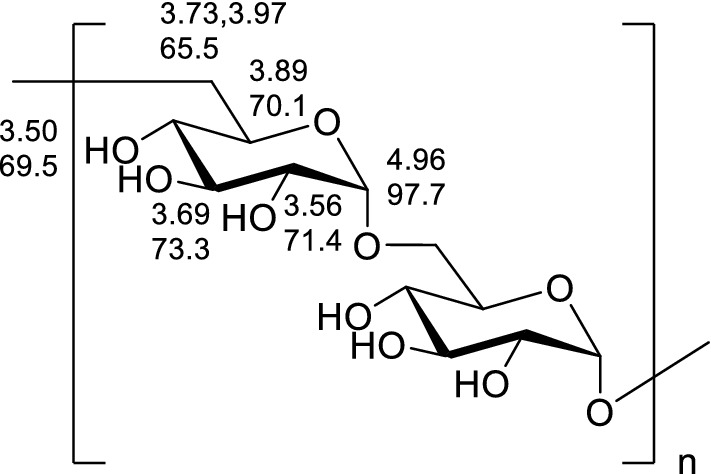
Structure of CPP 2–4.

The structure of CPP 2–4 was also confirmed by ^1^H-NMR and ^1^H–^1^H COSY, ^13^C-NMR, HSQC, and HMBC, with the results shown in Figs. 8, 9, 10 and 11. The ^13^C-NMR showed six carbon signals of glucose, δ_C_ 97.65, 73.35, 71.35, 70.13, 69.47, and 65.48 (see Table 4). The ^1^H NMR showed a terminal proton signal δ_H_ 4.96 (J = 2.5 Hz) in Table 4. Therefore, the conformation of glucose was determined to be α-type by the correlation between proton coupling constant. By analyzing the HMBC, we could see that H-1 was related to C-6, suggesting that the connection mode of the polysaccharide was 1 → 6 (see Fig. 12) ^37,38^.

**Table 4:** ^1^H and ^13^C NMR data for polysaccharide CPP 2–4 (in D_2_O solvent).

Position	δ_H_	δ_C_
1	4.96 (d, J = 2.5 Hz)	97.65
2	3.69 (d, J = 9.4 Hz)	73.35
3	3.56 (d, J = 9.8 Hz)	71.35
4	3.89 (d, J = 8.0 Hz)	70.13
5	3.50 (t, J = 9.4 Hz)	69.47
6	3.97 (d, J = 7.1 Hz) 3.73 (d, J = 12.5 Hz)	65.48

### Bioactivity of CPP 2–4

**Figure 13:**
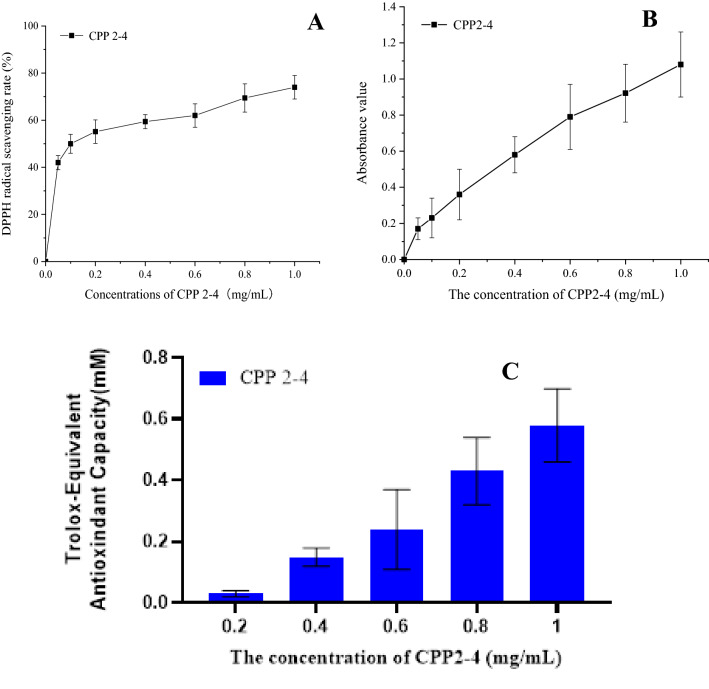
Antioxidant activity of CPP 2–4.

The DPPH free-radical scavenging ability of CPP 2–4 increased with increasing concentration, as shown in Fig. 13(A). The IC_50_ value of CPP 2–4 was 0.105 mg/mL, indicating that CPP 2–4 had a good ability to scavenge DPPH free radicals. As shown in Fig. 13(B), the total antioxidant capacity of CPP 2–4 increased with increasing concentration in the range of 0.05–1 mg/mL. As shown in Fig. 13(C), when the concentration of CPP 2–4 increased within 0.2–1 mg/mL, the TEAC value also increased, indicating that CPP 2–4 had a good ability to scavenge ABTS free radicals. The detailed data were shown in Table S5.

**Figure 14:**
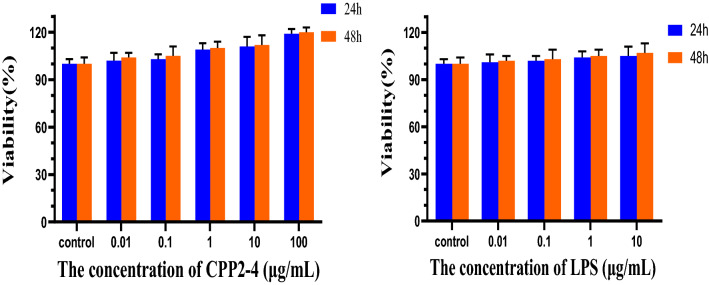
Viability of different concentrations of CPP2-4 and LPS.

The effect of CPP 2–4 on the proliferation of RAW264.7 cells is shown in Fig. 14. CPP 2–4 had no cytotoxic effect within 48 h when the concentration was 0.01–100 μg/mL. Similarly, the LPS was not toxic in the range of 0.01–10 μg/mL. Therefore, the LPS was relatively safe to establish inflammatory patterns on RAW264.7 cells. The detailed data were shown in Tables S6–7.

**Figure 15:**
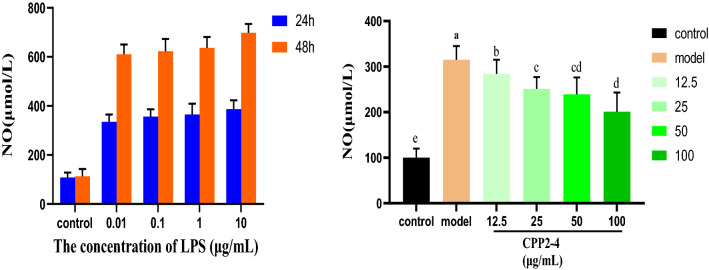
NO content in RAW246.7 cells.

Figure 15 shows the amount of NO released by LPS in RAW264.7 cells within 24 and 48 h. To avoid excessive LPS concentration causing cellular endotoxin tolerance, 1 μg/mL and 24 h were selected as conditions to establish the subsequent cell inflammation model^39^. Different concentrations of CPP 2–4 were administered to observe their inhibition of LPS-induced inflammation. The results showed that the NO content released by the culture medium was significantly lower than that of the model group (*, *P* < 0.05; **, *P* < 0.01). With increasing CPP 2–4 concentration, the NO content in the culture medium gradually decreased, indicating that the inhibition of NO release in RAW264.7 cells was dose-dependent. The detailed data were shown in Tables S8–9. When the membrane recognition receptor on the surface of macrophage RAW264.7 cells with immune function is stimulated by LPS, the inflammatory pathway is activated, and a large amount of inducible nitric oxide synthase (iNOS) is produced, resulting in the production of NO. NO is the main mediator in oxidative stress response, and can participate in and aggravate the inflammatory response. Therefore, in this study, the NO content in the macrophage RAW264.7 cell culture medium was determined, initially revealing that CPP 2–4 had a protective effect on inflammation, with its ability to inhibit NO release becoming stronger with increasing concentration.

## Conclusions

In this study, an efficient and rapid ATPS method was used to extract CPP for the first time. Single factor investigations combined with RSM were used to obtain optimized process conditions, as follows: Ammonium sulfate concentration, 17%; ethanol concentration, 30%; extraction temperature, 40 °C; pH 6. Under these conditions, the polysaccharide yield reached 31.57%, representing a major breakthrough. After dialysis and drying, two parts of polysaccharides were obtained in the top and bottom phases. As the bottom phase showed better antioxidant activity, it was subjected to further separation and purification to obtain homogeneous polysaccharide CPP 2–4. Study of the chemical properties and structural characteristics determined that CPP 2–4 was a dextran with a molecular weight of 3.9 × 10^4^ kDa, as isolated from *C. pilosula* for the first time. Activity studies showed that the IC_50_ value of CPP 2–4 for scavenging DPPH free radicals was 0.105 mg/mL, while the FRAP and ABTS’s results also indicated that CPP 2–4 had high antioxidant activity. Moreover, the RAW264.7 cells experiment initially revealed that CPP 2–4 had a protective effect on inflammation, which can inhibit NO release becoming stronger with increasing concentration. This paper provides a basis for the study of polysaccharide composition in *C. pilosula*, a reference for the extraction of polysaccharides in other medicinal materials, and data support for the medicine and food homology of *C. pilosula*.

## Supplementary Information

Supplementary Information.

## Data Availability

All data generated or analysed during this study were included in this published article [and its supplementary information files].
