# Supplementary material for: Extraction, structural characterization, and biological activities of a new glucan from Codonopsis pilosula
Source: Sci Rep. 2023 Mar 18;13:4504. doi: 10.1038/s41598-023-31660-2 (PMC10024767; doi:10.1038/s41598-023-31660-2)
Supplement: Supplementary file 1 — Supplementary Information. [file 41598_2023_31660_MOESM1_ESM.docx]

**Supplementary materials**

**1.Optimization of CPP extraction conditions**

**Table S1 Effect of (A) ammonium sulfate concentration on polysaccharide extraction yield in the ATPS method.**

| Factor | Conditions | Yield% | | Yield% | |
| --- | --- | --- | --- | --- | --- |
|  |  | Top phase | SD | Bottom phase | SD |
| (NH_4_)_2_SO_4_ (%) | 13 | 6.78 | 0.32 | 11.00 | 0.44 |
|  | 14 | 11.08 | 0.56 | 13.40 | 0.53 |
|  | 15 | 11.80 | 0.67 | 14.74 | 0.76 |
|  | 16 | 11.94 | 0.94 | 15.67 | 0.52 |
|  | 17 | 12.98 | 0.86 | 15.96 | 0.74 |
|  | 18 | 12.68 | 0.62 | 14.97 | 0.56 |

**Table S2 Effect of (B) extraction temperature on polysaccharide extraction yield in the ATPS method.**

| Factor | Condition | Yield% | | Yield% | |
| --- | --- | --- | --- | --- | --- |
|  |  | Top phase | SD | Bottom phase | SD |
| Temperature (%) | 10 | 2.68 | 0.52 | 8.18 | 0.53 |
|  | 20 | 3.54 | 0.64 | 10.73 | 0.75 |
|  | 30 | 7.50 | 0.93 | 14.64 | 0.51 |
|  | 40 | 9.33 | 0.87 | 17.83 | 0.73 |
|  | 50 | 6.75 | 0.69 | 15.89 | 0.51 |

**Table S3 Effect of (C) ethanol concentration on polysaccharide extraction yield in the ATPS method.**

| Factor | Condition | Yield% | | Yield% | |
| --- | --- | --- | --- | --- | --- |
|  |  | Top phase | SD | Bottom phase | SD |
| Ethanol (%) | 22.5 | 8.99 | 0.54 | / | 0.51 |
|  | 25 | 9.51 | 0.62 | 15.18 | 0.45 |
|  | 27.5 | 9.98 | 0.98 | 15.42 | 0.59 |
|  | 30 | 10.51 | 0.81 | 16.35 | 0.76 |
|  | 32.5 | 10.37 | 0.62 | 16.15 | 0.55 |

**Table S4 Table 3 Effect of (C) ethanol concentration on polysaccharide extraction yield in the ATPS method.**

| Factor | Condition | Yield% | | Yield% | |
| --- | --- | --- | --- | --- | --- |
|  |  | Top phase | SD | Bottom phase | SD |
| pH value | 3 | 2.01 | 0.57 | 4.35 | 0.56 |
|  | 4 | 6.35 | 0.63 | 10.61 | 0.35 |
|  | 5 | 12.90 | 0.65 | 14.65 | 0.59 |
|  | 6 | 13.42 | 0.81 | 15.25 | 0.43 |
|  | 7 | 13 | 0.65 | 15.5 | 0.53 |

**2. The bioactivity of CPP 2-4**

**Table S5 The antioxidant activity of CPP 2-4**

| Concentration of CPP 2-4 (mg/mL) | DPPH radical scavenging rate (%) | SD | Absorbance value | SD | Trolox-Equivalent Antioxindant Capacity (mM) | SD |
| --- | --- | --- | --- | --- | --- | --- |
| 0 | 0 | 0 | 0 | 0 | 0 | 0 |
| 0.05 | 42.01 | 3.23 | 0.17 | 0.06 | / | / |
| 0.10 | 49.96 | 4.14 | 0.23 | 0.11 | / | / |
| 0.20 | 55.13 | 5.64 | 0.36 | 0.14 | 0.03 | 0.01 |
| 0.40 | 59.39 | 3.24 | 0.58 | 0.10 | 0.15 | 0.03 |
| 0.60 | 61.99 | 5.17 | 0.79 | 0.18 | 0.24 | 0.13 |
| 0.80 | 69.43 | 6.08 | 0.92 | 0.16 | 0.43 | 0.11 |
| 1.00 | 73.98 | 5.13 | 1.08 | 0.18 | 0.58 | 0.12 |

**3. The anti-inflammatory action of CPP 2-4**

**Table S6 Viability of different concentrations of CPP 2-4**

| Condition | 24 h | | 48 h | |
| --- | --- | --- | --- | --- |
|  | Viability (%) | SD | Viability (%) | SD |
| control | 100 | 2.98 | 100 | 3.86 |
| 0.01 | 102 | 4.67 | 104 | 3.48 |
| 0.1 | 103 | 3.34 | 105 | 5.96 |
| 1 | 109 | 4.12 | 110 | 4.90 |
| 10 | 111 | 5.99 | 112 | 6.18 |
| 100 | 119 | 2.89 | 120 | 3.16 |

**Table S7 Viability of different concentrations of LPS**

| Condition | 24 h | | 48 h | |
| --- | --- | --- | --- | --- |
|  | Viability (%) | SD | Viability (%) | SD |
| control | 100 | 3.02 | 100 | 4.56 |
| 0.01 | 101 | 5.28 | 102 | 3.12 |
| 0.1 | 102 | 3.31 | 103 | 6.13 |
| 1 | 104 | 4.13 | 105 | 4.23 |
| 10 | 105 | 6.09 | 107 | 6.19 |

**Table S8 The NO content in RAW246.7 cells of LPS**

| Concentration of LPS (μg/mL) | 24 h | | 48 h | |
| --- | --- | --- | --- | --- |
|  | NO content (μmol/L) | SD | NO content (μmol/L) | SD |
| control | 108 | 20.12 | 113 | 30.09 |
| 0.01 | 335 | 30.31 | 610 | 40.13 |
| 0.1 | 356 | 31.11 | 623 | 50.21 |
| 1 | 365 | 44.24 | 636 | 45.15 |
| 10 | 387 | 36.15 | 698 | 36.27 |

**Table S9 The NO content in RAW246.7 cells of CPP 2-4**

| Different groups (μg/mL) | NO content (μmol/L) | | SD |
| --- | --- | --- | --- |
| control | 100 | 20.12 | |
| model | 315 | 30.36 | |
| 12.5 | 284 | 31.23 | |
| 25 | 251 | 26.37 | |
| 50 | 239 | 37.18 | |
| 100 | 201 | 42.29 | |
